# DNA Damage Response in Hematopoietic Stem Cell Ageing

**DOI:** 10.1016/j.gpb.2016.04.002

**Published:** 2016-05-21

**Authors:** Tangliang Li, Zhong-Wei Zhou, Zhenyu Ju, Zhao-Qi Wang

**Affiliations:** 1 Institute of Aging Research, School of Medicine, Hangzhou Normal University, Hangzhou 311121, China; 2 Leibniz Institute on Aging – Fritz Lipmann Institute (FLI), Jena D-07745, Germany; 3 Faculty of Biology and Pharmacy, Friedrich-Schiller University of Jena, Jena D-07745, Germany

**Keywords:** Hematopoietic stem cells, DNA damage response, Epigenetics, Ageing, P53

## Abstract

Maintenance of tissue-specific stem cells is vital for organ homeostasis and organismal longevity. **Hematopoietic stem cells** (HSCs) are the most primitive cell type in the hematopoietic system. They divide asymmetrically and give rise to daughter cells with HSC identity (self-renewal) and progenitor progenies (differentiation), which further proliferate and differentiate into full hematopoietic lineages. Mammalian **ageing** process is accompanied with abnormalities in the HSC self-renewal and differentiation. Transcriptional changes and epigenetic modulations have been implicated as the key regulators in HSC **ageing** process. The **DNA damage response** (DDR) in the cells involves an orchestrated signaling pathway, consisting of cell cycle regulation, cell death and senescence, transcriptional regulation, as well as chromatin remodeling. Recent studies employing DNA repair-deficient mouse models indicate that DDR could intrinsically and extrinsically regulate HSC maintenance and play important roles in tissue homeostasis of the hematopoietic system. In this review, we summarize the current understanding of how the DDR determines the HSC fates and finally contributes to organismal **ageing**.

## Introduction

In adult animals, tissue homeostasis is maintained by a hierarchy of different types of cells, ranging from tissue-specific stem cells, progenitors, to somatic cells with different functions [1]. Stem cells are the most primitive cell population in a specific tissue, which on the one hand self-renew to sustain the stem cell pool, and on the other hand differentiate to generate their somatic progenies [1], [2]. Dysregulation of self-renewal and differentiation of tissue-specific stem cells compromises the stem cell function, resulting in loss of tissue maintenance and organismal ageing [1], [3], [4].

**Figure 1:**
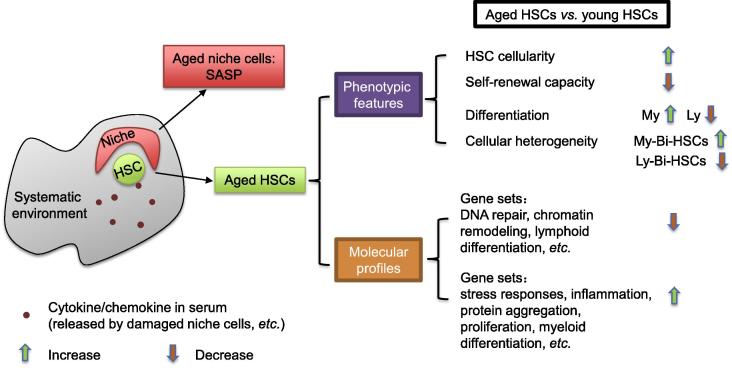
Characteristics of aged HSCs HSCs from aged mice show distinct phenotypic features and molecular profiles as compared to HSCs in young animals. The HSC ageing is a combinatorial effect from intrinsic determinants and extrinsic signals, such as niche cells and systematic environmental factors. HSC, hematopoietic stem cell; SASP, senescence-associated secretory phenotype; My, myeloid; Ly, lymphoid; My-Bi-HSC, myeloid-biased HSC; Ly-Bi-HSC, lymphoid-biased HSC.

Among all types of tissue-specific stem cells, hematopoietic stem cell (HSC) is considered as the prototype to study the functions of genes of interest in adult stem cell self-renewal and maintenance, as well as their roles in physiological ageing [5], [6]. Under unperturbed conditions, HSCs reside within their niches (bone marrow stromal cells) and are exposed to systematic environments consisting of cytokine, chemokine (Figure 1), and other factors [7]. The advantages of using the HSCs as the model to study stem cell ageing are mainly due to: (1) well-defined HSCs and their progenies with combinations of cell surface markers; (2) a panel of sophisticated *in vitro* assays to verify the HSC functions; and (3) the adoptive HSC transplantation assay as a gold standard to test stem cell functions [5]. Using naturally-aged wild type mice and genetically-modified premature ageing mouse models [8], [9], [10], [11], [12], [13], intrinsic and extrinsic factors contributing to the HSC ageing start to be unraveled [4], [14], [15], [16]. Among them, cell cycle regulators, transcriptional factors, epigenetic modulators, and metabolic pathways have been implicated as important regulators for HSC self-renewal and maintenance during ageing process [10], [12], [17], [18], [19], [20], [21], [22], [23].

DNA lesions in cells originate from endogenous cellular activities, such as DNA replication and mitochondrial respiration, as well as exogenous stimuli, such as therapeutic drugs against cancers and medical exposure to irradiation, posing direct threats to the integrity of the cellular genetic information [24], [25], [26]. If these DNA lesions could not be handled well, they will compromise cellular viability and drive the tumor formation [27], [28]. When it comes to the HSCs, improper repair of DNA lesions could negatively regulate the HSC maintenance and lead to HSC ageing [4], [8], [26]. Here, we concisely discuss the signatures defining “aged HSCs” and the role of genomic stability in HSC ageing.

## Characteristics of HSCs in ageing hematopoietic system

Compared to the young individuals, the frequency (percentage of HSCs within bone marrows) and absolute numbers of HSCs, which are phenotypically designated with defined surface markers, increase in naturally-aged individuals of mice and humans (Figure 1) [8], [29], [30]. However, HSCs in aged mice are defective in the self-renewal capacity [31]. The adoptive bone marrow transplantation assay is the “gold standard” to investigate the HSC functionality. Upon transplantation, HSCs are forced to enter the cell cycle and differentiate into different hematopoietic lineages [32]. The sequential transplantation with the HSCs from the primary transplantation could be further employed to test the robustness of HSCs in self-renewal. During the serial transplantation, HSCs get exhausted and step into an “aged” status [12], [33]. Using this serial adoptive transplantation assay, aged HSCs (HSCs from aged mice) showed limited repopulation ability to replenish the hematopoietic system in bone marrow-ablated congenic mice [12], [29]. The HSC transplantation assay indicates that the aged HSCs, in addition to a homing defect (a failure of transplanted donor HSCs trafficking to and engrafting in recipient bone marrows), only represent around 25% efficiency of HSCs from young animals [29].

Furthermore, aged HSCs have differentiation defects as well (Figure 1). Peripheral blood (PB) from aged mice contains a relative higher proportion of myeloid cells, such as Mac1 ^+^ and Gr1^+^ hematopoietic cells, as compared to the PB from young animals [29], [34], [35], which could be attributed to the higher proportion of myeloid progenitors generated in the bone marrow of aged mice [36]. The biased myeloid hematopoiesis in the aged mice is detrimental to hematopoietic system functions since the dysregulated output of lymphoid and myeloid cells would compromise the immunological response upon injury or infection in the aged animals and further promote ageing. This skewed differentiation is cell-autonomous, since transplanting aged HSCs to young mice could recapitulate the phenotypes of “ageing” hematopoietic compartments in these recipient mice [34], [36]. The increased ratio of myeloid *vs*. lymphoid hematopoietic cells in ageing is further attributed to altered heterogeneity in HSC compartments during the ageing process [37], [38]. Based on their differentiation capabilities, HSCs are further divided into lymphoid-biased HSCs (Ly-Bi HSCs), myeloid-biased HSCs (My-Bi HSCs), and balanced HSCs [17], [37]. The composition of HSC pools is shifted from Ly-Bi HSCs toward My-Bi HSCs during ageing.

In addition to the aforementioned phenotypically-defined characteristics, aged HSCs are distinct from young HSCs due to their unique transcriptomic and epigenomic features [39], [40], [41]. Aged HSCs are implicated with marked increase in the expression of genes involved in stress responses, inflammation, and protein aggregation, while the expression of factors responsible for DDR and chromatin remodeling is reduced (Figure 1) [40]. Accordingly, aged HSCs accumulate DNA lesions [8], are defective in protein homeostasis, and exhibit abnormal epigenetic landscapes on DNAs and histones [9], [39], [40]. DNA methylation is enriched specifically on the promoter regions of lymphoid and erythroid lineage genes [9]. On the contrary, promoters of genes responsible for the myeloid lineages exhibit reduced DNA methylation. This finding correlates with the skewed hematopoietic lineage output in aged mice [8], [36], [42]. Furthermore, hypo-methylated cysteines and active chromatin markers, such as H3K9me3 and H3K27me3, are enriched in the promoter regions of genes in the Gene Ontology categories of cell adhesion, proliferation, and ribosome, which are expressed higher in the aged HSCs than in the young ones [39]. Such transcriptional and epigenetic alterations could partially explain the phenotypic characteristics of aged HSCs, such as an increased mobilization, reduced homing ability, and loss of quiescence [29], [39].

## DNA damage accumulation in HSC ageing

In somatic cells, loss of genomic integrity compromises the cellular viability and threatens the genetic information passage from parent cells to daughter cells [43]. Accumulation of genomic instability has been implicated in the hematopoietic malignancy, which could be derived from transformed HSCs [26], [28]. DNA lesions initiate DDR and induce chromatin remodeling, epigenetic modification, as well as transcriptional regulation, which consequently activate a series of cellular responses including DNA repair, cell cycle checkpoint, cellular senescence, and cell death [24], [25], [26], [43]. All of these pan-genome, epigenome, and transcriptome modifications definitely generate systematic outcome to shape the dynamics of the HSCs in the context of self-renewal and differentiation [44], [45].

The first direct link of DNA damage and HSC ageing comes from the analysis of double strand breakage (DSB) marker γ-H2AX in murine HSCs [8]. Rossi and colleagues investigated the DNA damages inside aged murine HSCs and noticed that aged HSCs accumulate high levels of DNA DSBs [8]. However, a recent study suggested that those γ-H2AX marked “DSB foci” may not be real DNA breaks. The “DSB foci” are nucleolar-associated and represent the residual replication stress during the HSC cycling [46]. The “γ-H2AX foci” severs as the chromatin repressive marker for the silencing of rDNA transcription, which compromises the ribosome biogenesis in aged HSCs [46]. Interestingly, Beerman et al. used the alkaline comet assay, which is extensively used in the field of DNA repair as the indicator for the DSBs and single strand breaks (SSBs) [47], [48], to compare the DNA damages in young and aged quiescent HSCs. As a result, they noticed that aged HSCs have a high degree of DNA breaks, as indicated with increased “Olive tail moment” [9], [48]. Similar to the murine HSCs, γ-H2AX antibody staining on human CD34^+^ HSCs and hematopoietic progenitors reveals an significant accumulation of DSBs during normal ageing process [49]. These data indicate that murine and human HSCs experience similar biological processes, namely genomic instability, during physiological ageing.

How are these DNA breaks generated in HSCs under physiological conditions during the ageing process? Reactive oxygen species (ROS) generated from metabolic pathways in quiescent HSCs and replication errors during HSC proliferation could be the threats to genome integrity in HSCs [33], [50], [51]. Using different mouse models that harbor deficiencies in DNA repair pathways, it is found that loss of DNA repair factors results in accumulation of DNA damages in HSCs and severely- compromised capabilities of HSCs for self-renewal and differentiation under physiological conditions [8], [11], [52], [53], [54]. For examples, knockout mice with defects in DNA DSB repair and quenching the ROS (such as *Atm*^-/-^ mice) or in resolving the replication fork stalls (knockout of Fancd2 pathway members) are ageing-prone and show defective hematopoiesis [51], [55], [56]. These findings strongly indicate that the proper repair of DNA damage is important for the maintenance of HSCs and protects against functional decline of HSCs during ageing [4], [8], [26], [45].

## DNA repair pathway choices in HSCs

In order to fix DNA breaks, cells are equipped with different repair mechanisms or repair factors [24], [43]. The choice of pathways to repair a DNA lesion is highly dependent on the cell cycle phase and/or the physiological status of a cell [25], [57], [58]. Our knowledge of DNA repair in cell cycle stems from studies on the cycling somatic cells. Intriguingly, adults HSCs reside in quiescent status (G0) [14]. Two repair scenarios have been proposed to repair ionizing radiation (IR)-generated DSBs in HSCs in G0 phase [59], [60]. Passegué and her colleagues found that quiescent HSCs use the same repair program as in G1 phase of somatic cells, *i.e.*, non-homologous end joining (NHEJ) pathway to repair the IR-generated DNA lesions [60]. In this scenario, upon DSB induction, protein complex comprising MRE11/RAD50/NBS1 is recruited to the DSB sites and activates ATM kinase, which phosphorylates MDC1/H2AX/53BP1/SMC1/KAP1 to alter the chromatin status around the DSBs and CHK2/p53 to initiate the cell cycle checkpoints and/or cell death signaling pathways [24], [61], [62], [63]. In addition, MRE11 nuclease resects the DNA strand at DSBs to generate micro-homology to facilitate the repair process [62], [64], [65]. This repair pathway is considered as a low fidelity repair choice, since abnormal chromosome fusions, loss of genetic material around the breakage sites, and accumulation of genetic mutations could happen following the repair [63]. Indeed, Passegué and her colleagues found that 53BP1 foci (a marker of NHEJ) rather than RAD51 (a marker of homologous recombination, HR) were prominently evident in IR-treated quiescent HSCs [60]. Consequently, chromosome analysis with spectral karyotyping (SKY) on the hematopoietic progenitors derived from IR-irradiated HSCs reveals a great increase in genome instabilities, including chromosome fusions [60]. However, this repair pathway could not effectively explain the accumulation of DNA break marker γ-H2AX in naturally-aged HSCs, because ligation of DNA breaks by NHEJ quenches the DDR signaling and thereby generates γ-H2AX-free HSCs [8], [42]. It has been proposed that quiescence is a cellular status when HSC loses its stringent control of repair machineries [42], [48]. Beerman et al. conducted the *in vitro* short-term culture of isolated quiescent HSCs [48]. After 24 h, a significant reduction in γ-H2AX-marked DSBs was noticed when the HSCs enter the cell cycle [48], suggesting that proliferating HSCs repair DSB better. G0 HSCs apparently express low levels of DDR genes as compared to proliferating HSCs (such as fetal liver HSCs) and progenitors [48]. In this way, DNA damage signaling may be attenuated in quiescent HSCs, which is consistent with the previous finding on accumulation of DNA breaks in aged HSCs [8], [42].

Once HSCs are mobilized and forced to enter cell cycle by *in vivo* administration of cytokine granulocyte-colony stimulating factor (G-CSF) or cultured *in vitro* in the presence of the stem cell factor (SCF), HSCs switch the repair mechanism from NHEJ toward HR [60]. In S/G2/M HSCs, MRN complex recruits ATM and resects the DSBs to generate the single strand overhangs, which can activate ATR/CHK1 kinase [63], [66]. RAD51 is then loaded onto the exposed single strands and forms DNA/protein filaments to initiate strand invasion into their homologous chromosomes [67]. As compared with NHEJ in G0/G1 cell cycle, HR is more stringent in keeping the genomic integrity. However, although HSCs can faithfully repair the DNA breaks when cycling [46], [48], [51], entry into cell cycle could be detrimental to the quality of HSCs because serial transplantation experiments in mice demonstrate that HSCs have limited replicative lifespan and multiple rounds of stress-induced HSC cycling can compromise self-renewal and differentiation capacity, leading to exhaustion of the HSC pool [12], [46].

## p53-p21/PUMA pathway in HSC cell fate determination

**Figure 2:**
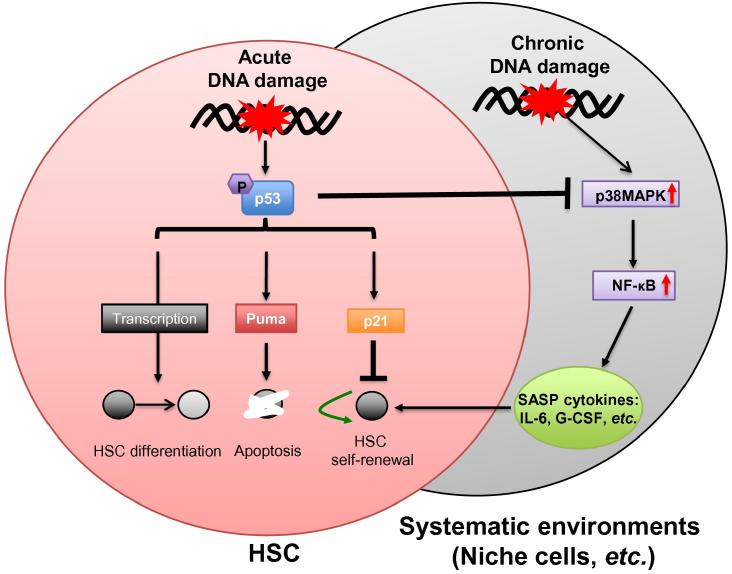
p53 signaling in HSC fate determination toward ageing Hematopoietic stem cell fates are determined intrinsically with molecular events insides a HSC and extrinsically by its residing environments, including HSC niche cells and circulating serum factors*.* p53 signaling plays important roles in HSC fate determination upon DNA damage. After acute DNA damage induction, cell cycle arrest/senescence and cell death pathways medicated by p53-p21 and p53-PUMA determine the HSC pool dynamics. Transcriptional regulation by stabilized p53 exerts additional impact on the HSC transcriptome and leads to HSC differentiation, *etc.* Furthermore, senescent or heavily-damaged cells secrete pro-inflammatory cytokines (SASP cytokines) to drive HSCs into cycling, which consequently triggers HSC exhaustion. SASP is proposed to be dependent on p38MAPK-NF-κB signaling. In this scenario, p53 signaling plays beneficial roles in inhibiting the SASP by suppressing the p38MAPK activation and subsequent NF-κB transcriptional activation. HSC, hematopoietic stem cell; SASP, senescence-associated secretory phenotype; G-CSF, granulocyte-colony stimulating factor.

DNA damages exhaust HSCs in terms of self-renewal and differentiation by reducing the HSC pool (quantity) and compromising HSC stemness (quality) [26], [35], [45]. Upon DNA damage, cells engage a serial of downstream cellular events including cell cycle arrest, apoptosis, and transcriptional reprogramming [43]. Faithful DNA repair preserves the HSC genome integrity and sustains HSC stem cell identity in proper self-renewal and differentiation. However, depending on the repair efficiency for certain DNA lesions, HSCs undertake different fates toward permanent cell cycle arrest (senescence), cell death (HSC elimination) [68], [69], and even differentiation [70], [71], [72], [73]. As the HSC fate determinant, the p53 pathway has been well studied *in vivo* [74], [75]. The p53 pathway is transiently activated after a single dose of IR or can be constantly activated in HSCs by persistent DNA damages, such as critically shortened telomeres [60], [69], [76]. Downstream of p53 signaling, p53 trans-activates p21 to promote the cell survival by initiating cell cycle arrest for DNA repair or cellular senescence, while induced expression of the p53 upregulated modulator of apoptosis (PUMA) by p53 is responsible for cellular clearance (Figure 2) [13], [68]. Inhibition of either branch of p53-p21 or p53-PUMA benefits HSC self-renewal and maintenance in several cases of HSC ageing mice models [74], [77], [78], [79]. Complete p53 loss renders cytoprotective effects on IR-damaged HSCs [80] and promotes symmetric division of HSCs to expand the HSC pool [74], [81], [82]. However, these *p53*-null HSCs show defective differentiation and are tumor-prone, indicating that *p53* null compromises the HSC quality [74], [78]. In this regard, the balance of p21 and PUMA downstream of p53 signaling is essential for the maintenance of HSCs and hematopoietic system [75], [83].

Compared to hematopoietic progenitors, murine HSCs are resistant to acute DNA damage induction and prone to survival [60]. This is likely due to a high expression level of the pro-survival genes in murine HSCs [60]. In response to acute DNA damage, HSCs tend to be arrested and reside in the senescent status, suggesting that p53-p21 branch is activated in HSCs preferably to limit HSC self-renewal [77], [84]. Loss of p21 in the mouse model with persistent DNA damage (the 3rd generation of *Terc*^−/−^ mice; G3 *Terc*^−/−^) with critically-shortened telomeres could partially rescue ageing phenotypes by improving the repopulation capacity and self-renewal of HSCs [77]. Furthermore, activated p53-PUMA branch is responsible for the HSC death upon lethal dose of γ-irradiation (10 Gy), since loss of PUMA protects the HSCs and extends the lifespan of irradiated mice [85]. The constitutive activation of p53 signaling in mice lines expressing p53 phosphorylation mutations (T21D and S23D), a C-terminal truncated p53 allele, or other gene mutations confers premature ageing of hematopoietic systems [13], [86], [87]. Genetic ablation of *Puma* restores the viability of HSCs, indicating p53-PUMA limiting the HSC pool size [85], [86]. These data point to a promising therapeutic strategy to protect HSCs and prolong healthy lifespan with p21 or PUMA inhibitors. However, it is of note that *p21*-null HSCs exhibit self-renewal defects in serial transplantation assay, while *Puma*-null HSCs are superior to their wild type controls [86], [88]. These findings indicate that p53-p21 and p53-PUMA have differential roles in mediating HSC fates and ageing due to different extents of DNA lesions (Figure 2).

## Persistent DNA damage creates a pro-ageing environment for HSCs

Intrinsic defects in repairing DNA damages and their contributions to compromised HSC self-renewal and ageing process have been extensively studied in recent years. However, ageing environments, such as mis-regulated cytokine factor secretion and altered stem cells niches, can all affect HSC maintenance (Figure 1) [4], [76], [89], [90]. An interesting example of the environmental impact on the HSC self-renewal and differentiation comes from the parabiosis assay by surgical connection between young and aged mice with the circulatory blood system [91], [92]. In this assay, young and aged HSCs are unanimously exposed to a common systematic environment, such as serum factors and osteoblast niches. Multi-organ analysis shows that the interconnection of young and aged mice significantly improves tissue homeostasis including HSCs and hematopoietic system of the aged mice. These data strongly indicate that a young systematic environment can rejuvenate the aged HSCs [91], [92]. Although the search for the key factors in the systematic environment that contribute to the ageing and rejuvenation of stem cells is still ongoing, these findings conceptually prove that the HSCs, in addition to the intrinsic regulation, could be functionally modulated by the environmental cues. On the other hand, parabiosis assay highlights a novel concept that HSC ageing could be delayed or partially reversed by rejuvenating serum factors [69], [91], [92].

Does DNA damage generate a systematic change and promote the HSC ageing? The analysis of HSCs from G3 *Terc*^−/−^ mice unveils some hints on this question. The critically-shortened telomeres in G3 *Terc*^−/−^ mice can be recognized as persistent DNA breaks, which constantly activate the p53-p21/PUMA pathway in tissue-specific stem cells and their somatic progenies [77], [89], [93]. Ju et al. analyzed the interplay between HSCs and their niches, *i.e.*, mesenchymal stem cell (MSC)-derived bone marrow stromal cells. In G3 *Terc*^−/−^ mice, the functionality of both MSCs and bone marrow stromal cells is compromised. In addition, the high level of G-CSF cytokine in the G3 *Terc*^−/−^ mice serum significantly reduces the engraftment of HSCs in bone marrow niches [76]. G-CSF inhibition leads to the improved engraftment and functionality of HSCs. These findings suggest that a cellular response from those “damaged” cells with persistent DNA lesions caused by telomere shortening could alter the local environment (such as HSC niches) or systematic environment (*i.e.*, cytokines or chemokines in serum) and confer a deleterious effect on self-renewal and maintenance of HSCs [69], [76], [89].

How persistent DNA damage signaling can change the systematic environment? One possibility could be the senescence-associated secretory phenotype (SASP) [94], [95]. Campisi and colleagues found that senescent cells, although permanently arrested in cell cycle, is metabolically active in producing inflammatory factors (SASP cytokines), such as IL-6, IL-10, INF γ, and G-CSF [94]. Furthermore, not only senescent cells, but also cells with persistent DNA damages, exhibit SASP and secrete pro-inflammatory cytokines to change systematic environment in the animal tissues [96]. Activated NF-κB signaling has been implicated in SASP, since inhibition of NF-κB signaling by knocking down of p65 greatly alleviates the expression of SASP cytokines [97], [98], [99]. Furthermore, activation of p38MAPK kinase activity by various stimuli promotes SASP induction. p38MAPK sits upstream of NF-κB signaling and regulates the NF-κB activity. Interestingly, although p53 is not required for initiating SASP, p53 restrains SASP once the cellular senescence is established, since *p53*-null cells show enhanced expression of SASP cytokines [99]. The inhibitory effects of SASP by p53 could be attributed to the fact that p53 restrains p38MAPK activity via its DDR-independent activity. In this sense, p53 DDR-independent signaling may provide protective roles in maintaining HSC homeostasis by inhibiting SASP in the systematic environment of hematopoietic system (Figure 2).

The secreted inflammatory factors from these cells with persistent DNA damages, may be detrimental to the HSC self-renewal and maintenance. For example, G-CSF mobilizes the HSCs out of bone marrow niches and impairs their engraftment [76], [100]. IL6, INFα, and INF γ have been implicated in promoting the cell cycle entry of HSCs, resulting in HSC exhaustion toward ageing [90], [101]. Tissues in aged animals are enriched in senescent somatic cells that contain persistent DNA damages. Furthermore, ageing process positively correlates with increased inflammatory responses [102], which may further ameliorate the HSC maintenance and promote ageing [103]. Recent studies indicate that interfering with SASP by blocking the cytokine production pathway or eliminating cytokine-producing cells in tissues greatly improves the tissue function and animal lifespan. Clearance of p16Ink4a-positive senescent cells in progeroid BubR1 mutant mice could substantially delay the onset of ageing phenotypes and even rejuvenate the ageing tissues when such cellular clearance was applied in late-life of BubR1 mutants [104]. Furthermore, Chang et al. employed ABT263, a specific chemical inhibitor for Bcl-2 and Bcl-xL, to induce the apoptosis of senescent HSCs. They found that ABT263 treatment restores the functionality of HSCs in the sub-lethally irradiated wild type mice and naturally-aged mice, thus greatly improving the healthy lifespan of mice [105]. The data further confirm the assumption that senescent cells with persistent DNA damage can establish a systematic environment driving HSC ageing.

## Conclusions and perspectives

Integrities of HSC pool and HSC quality are considered as the key factors contributing to the organismal ageing in mammals [4], [29]. DDR plays essential roles in the maintenance of these two HSC features [8], [44], [45], [52], [68]. Deficiency in DNA repair results in the accumulation of unrepaired DNA breaks in aged HSCs, while persistent DNA breaks in hematopoietic cells and HSC niches create a pro-inflammatory environment to promote HSC entry into cell cycle and proliferation, which consequently exhausts HSCs [69]. It would be very tricky to experimentally modify those ageing HSCs with genetic approaches in order to achieve the better DNA repair and functional improvement. Instead, using bio-active cytokines to interfere with the systematic ageing environment or using small chemical molecules to specially remove the “aged” HSCs would be the reliable and practical strategies to rejuvenate the ageing HSCs and prolong the healthy lifespan of mammals [105], [106].

## Competing interests

The authors declare that they have no competing interests.
